# Skin Barrier in Normal and Allergic Horses: What Do We Know?

**DOI:** 10.3390/vetsci12020091

**Published:** 2025-01-24

**Authors:** Rosanna Marsella

**Affiliations:** Department of Small Animal Clinical Sciences, College of Veterinary Medicine, University of Florida, Gainesville, FL 32608, USA; marsella@ufl.edu

**Keywords:** horse, skin barrier, TEWL, pH, microbiome, allergies

## Abstract

Information on skin barrier in horses is limited. A study on the epidermal ultrastructure of normal and allergic horses documented disorganized amorphous intercellular lipids in the stratum corneum of allergic samples. These findings are similar to atopic canine and human skin. Currently, there is no published study comparing skin barrier function parameters between normal and allergic horses; thus, the functional implications of the ultrastructural changes are unknown. In normal horses, body location, gender, breed, and ambient conditions affect skin barrier parameters, such as Transepidermal Water Loss. Skin microbiome studies on normal horses have highlighted the importance of season and environmental conditions, since horses housed together share similar microbiomes. Skin dysbiosis and predominance of staphylococcus have been described in horses with pastern dermatitis. Transcriptomic studies of the epidermis of normal and allergic horses have found that lesional allergic skin has substantial transcriptomic differences when compared with healthy skin, namely downregulation of genes of tight junctions, keratins, and upregulation of serine proteases and IL-13. Keratinocytes harvested from horses with insect bite hypersensitivity show upregulation of IL-31 gene expression under stimulation. While more research is clearly needed, preliminary results seem to support skin barrier differences between normal and allergic horses.

## Simple Summary

Skin itself plays an important role in the development of diseases like eczema in both people and dogs. People with eczema have very permeable, sensitive skin that has less protection against the penetration of pollen and dust. The presence of “leaky” skin starts a cascade of events, leading to allergy development. Thus, products to keep the skin moist and “sealed” in eczema patients are very important. Horses suffer from allergies similar to the ones of people, but the role of the skin in the development of equine disease is largely unknown. This review focuses on the current level of knowledge on skin barrier function in normal and allergic horses. Based on the preliminary information, it appears that horses have many similarities to people: the structure of the skin itself in allergic horses is different from the one of normal horses, and it is very similar to the one of people with eczema. It is not known if the skin of allergic horses also absorbs more pollen or what is the best way to correct these abnormalities. More work is needed to explore skin barrier function in allergic horses with the goal of improving the treatment of this disease.

## 1. Introduction

The term skin barrier refers to the combination of physical, chemical, immunological, and microbiological barriers. While keratinocytes were historically viewed as an inert, merely physical barrier, it is now clear that they are much more than a physical barrier and that they are far from passive. They are instead active participants in the immune response to allergens and pathogens by releasing cytokines that shape the immune response to allergens; they release antimicrobial peptides that contribute to the host defense against pathogens, and they produce proteins and lipids critical for proper barrier [1,2].

Increasing attention is given to skin microbiome in health and disease, as an important component of the skin barrier. Skin microbiome shapes the immune response and boosts the chemical barrier of the skin by producing enzymes that increase free fatty acid production, thus contributing to the acidification of the pH of the skin. Thus, currently, the microbiome is included as one of the vital components of the skin barrier [3,4]. In healthy individuals, a high biodiversity of microbes is beneficial to physically occupy niches that otherwise may be available to pathogens. Microbiota is also required for proper keratinization and the sphingomyelinases produced by skin bacteria are important to process lamellar body lipids into ceramides, thus contributing to proper skin barrier function. These beneficial microbes produce antimicrobial peptides that inhibit pathogens; thus, any shift in the diversity of normal microbiomes can have profound consequences on the health and function of the skin.

**Figure 1:**
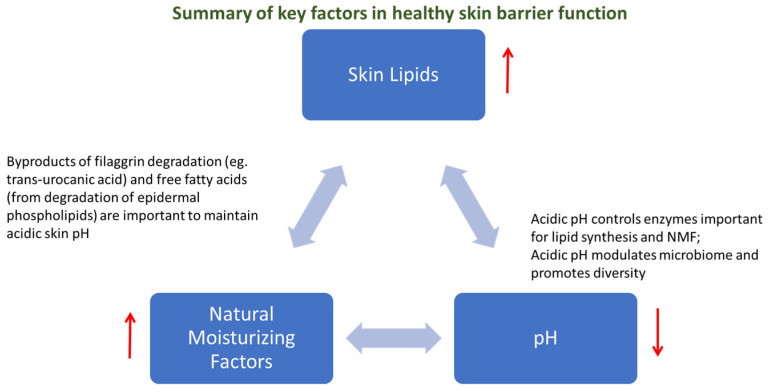
Summary of the key factors that play a role in maintaining a healthy skin barrier function and their relationships.

The key components that regulate skin barrier function can be summarized in three aspects: the stratum corneum lipids, the natural moisturizing factors (resulting from filaggrin metabolism), and an acidic pH. These factors are interconnected and tightly regulated in healthy skin. A decrease in the stratum corneum lipids leads to an increase in skin permeability, and a decrease in natural moisturizing factors leads to a decrease in hydration [5]. Changes in pH have profound effects on enzymes important for natural moisturizing factors production, lipid synthesis, and microbiome homeostasis [6]. For example, when pH increases, the synthesis of ceramides is decreased, proteolysis is increased, and the integrity of the stratum corneum is decreased (Figure 1).

Skin barrier function in the context of allergic skin diseases is a topic of much research in diseases like human and canine atopic dermatitis [7,8]. In both species, it is widely accepted that skin barrier dysfunction plays an important role in both the sensitization and the elicitation of clinical signs [9,10] and that skin barrier impairment is detectable in atopic individuals, and it is aggravated by inflammation [11,12]. Importantly, there is building evidence that epicutaneous sensitization to allergens not only affects skin reactivity but triggers a systemic TH2 response and is linked to reactivity in other organs, such as the lungs [13]. TH2 cells are distributed systemically through the circulatory system, where they activate eosinophils and promote eosinophil infiltration into inflamed tissues.

**Figure 2:**
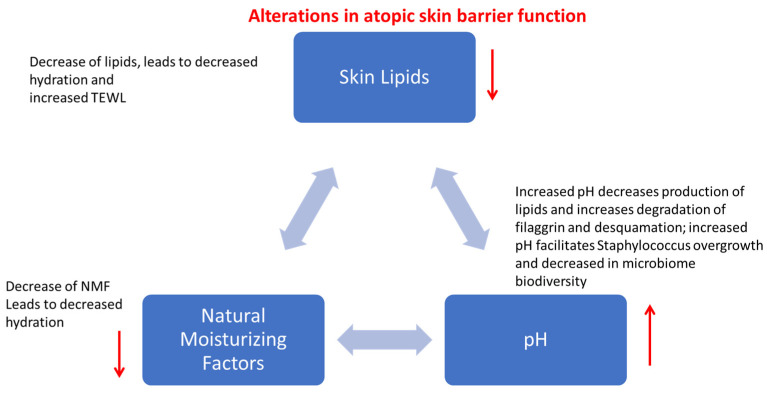
Summary of the main abnormalities seen in atopic skin and how changes in one parameter affect the others leading to a progressive vicious cycle of worsening of skin barrier function. TEWL: Transepidermal Water Loss.

In atopic skin, pH is increased [14,15], leading to decreased lipid synthesis and decreased natural moisturizing factors. The increased pH of allergic skin impairs the extrusion of lamellar bodies, which is important for lipid synthesis but also for the release of antimicrobial peptides, which ultimately facilitates the overgrowth of Staphylococcus. The dysbiosis due to Staphylococcal overgrowth further increases skin pH leading to vicious cycles of skin barrier worsening (Figure 2).

Information concerning the skin barrier in horses, particularly in equine allergic skin diseases, is still limited. The purpose of the current review is to summarize what is currently known about skin barriers in normal and allergic horses and what areas need future studies.

## 2. Skin Barrier Function in Normal Horses

Skin barrier function is often measured with biophysical parameters like Transepidermal Water Loss (TEWL), hydration, and pH. Transepidermal water loss has been traditionally used to assess the permeability of the skin and it has been documented that higher values are associated with increased vulnerability of the skin to allergens. This parameter is, however, temperamental, and extensive reports in human medicine have documented how TEWL can be variable [16], how it is affected by a variety of factors [17], and how it is critical for investigators measuring it to be knowledgeable and consistent in order to maximize the benefits of this measurement [18]. pH, on the other hand, seems to be the most consistent parameter [19,20]. In healthy horses, pH can significantly differ by day and by time of the day [21]. This finding was reported in a small study that considered five healthy mares and took measurements in different anatomical locations over the course of 5 days. Interestingly, in that pilot study, the anatomical location did not seem to affect the pH and there were no significant associations with ambient temperature and humidity. Consistently with other reports in people [16,17] on the variability of TEWL, the small study in normal horses [21] reported that TEWL significantly differed by day, time, and anatomical location. Transepidermal Water Loss negatively correlated with ambient humidity and positively correlated with temperature, regardless of the anatomical location. Thus, TEWL measures are affected by environmental conditions, and this is an important consideration when this parameter is compared between healthy animals and horses with allergic skin diseases in the future. While in dogs and people ambient conditions can be standardized and animals can be acclimatized prior to taking TEWL measurements, in horses, this is nearly impossible, as most of them are in some form of outdoor situation and measurements will not be taken from all horses enrolled in a study all on the same day and in the same location.

Transepidermal Water Loss is also affected by body location [22,23]. Lumbar areas have lower TEWL values, and the lips have the highest TEWL values. In terms of hydration, large variability was reported for the lip and the tightest values for the thorax [22]. pH does not appear to be significantly affected by anatomical location, although a large variability of values has been reported, especially in the pinnae [22]. More studies in various horse breeds and different anatomical locations are useful to evaluate the reliability of skin pH measurements.

Transepidermal Water Loss is also affected by breed [24]. When four breeds were studied (Felin ponies, Polish koniks, Polish cold-blooded horses, and Wielkopolska horses), an effect of the breed was found in the lumbar and inguinal region, while no other significant differences were found for the shoulder, lateral thorax, or pinnae. Thus, in the future, when normal and allergic horses are compared for TEWL, it will be important to be mindful of the anatomical location to minimize the effect of breed and/or to make sure that the normal and allergic groups are matched by breed.

Gender also affects the biophysical parameters of skin in normal horses [25]. More specifically, significant differences in TEWL values exist in the lips of mares compared with stallions and in stallions compared with geldings. Corneometry also showed significantly higher results in the neck region in mares compared with stallions. All these findings highlight the importance of matching populations for gender when normal and allergic horses are compared in the future. Importantly, the measurement of TEWL is also affected by the presence of the hair, with the highest variability found in unclipped areas and the most consistent values in clipped areas [26].

**Figure 3:**
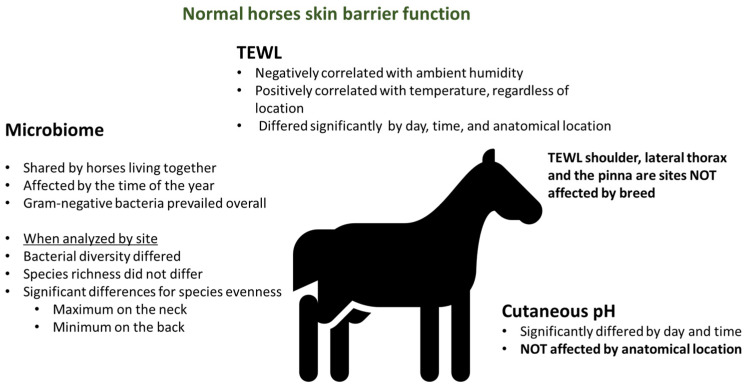
Summary of the current level of information on skin barrier parameters and microbiome in healthy horses.

The microbiome in healthy horses is another developing area of research where we are starting to gather some knowledge. In one study, 12 clinically healthy horses from the same farm were studied [27]. Samples were collected at four time points (winter, spring, summer, autumn), and the authors found that horses living together shared overlapping microbial populations. The bacterial diversity, richness, and evenness were not significantly different in the four body sites tested. The greatest variation in microbial populations was due to the time of the year. This effect was more profound than the body site and the individual horse. Thus, when future studies consider microbiomes in healthy and allergic horses, it will be important that subjects of healthy and diseased groups are housed on the same farm to control for environmental conditions and that they are all sampled at the same time of the year. Another study, which focused on female Shetland ponies kept under the same environmental conditions, showed that, when microbiome was analyzed in four different sites (neck, back, abdomen, pastern, muzzle), bacterial diversity differed, while species richness did not differ [28] according to the site. Results of the study indicate that Gram-negative bacteria overall prevailed in normal skin. Eighteen phyla, twenty-nine classes, and one hundred nineteen families were found. The most abundant phyla were Proteobacteria (approximately 30–40%), followed by Actinobacteriota (approximately 20%), Firmicutes (15–20%), Bacteroidota, and Deinococcota (each group approximately 10%). Species evenness varied by site, with maximum evenness found on the neck and the minimum one on the back, while richness and diversity of bacterial species had no major differences in the five regions examined. The mean relative abundance of bacterial genera was highest on the back and lowest on the abdomen. As far as the Gram-negative bacteria, it was considered that the original is environmental, which is confirmed by how horses living in the same environment share similar skin microbiomes [27]. The main findings regarding skin barrier function in normal horses are summarized in Figure 3. 

## 3. Skin Barrier in Horses with Skin Allergies

Currently, there is no study on skin pH and TEWL in horses with allergic skin disease. One initial report [29] described the ultrastructure of atopic equine skin and compared it to normal skin. In this report, the atopic horses were selected after having ruled out an insect allergy. Horses had seasonal signs and did not respond to an aggressive insect control program. One atopic horse also had signs of respiratory disease. Biopsies were taken from both lesional and non-lesional atopic skin and compared with site-matched skin biopsies of normal horses. The study was small with two horses in each group but it showed remarkable differences in the ultrastructure of the upper epidermis between atopic and normal horses. While normal horses showed nicely organized lipid lamellae, the atopic samples showed severely disorganized amorphous lipid material in the intercellular spaces of the stratum corneum of lesional atopic skin. Ultrastructural changes in non-lesional skin were also present but not as severe as seen in the lesional skin samples. Retention of lamellar bodies in the corneocyte of atopic horses was also documented, similar to what is reported in human atopic patients. The defect in the extrusion of the content of lamellar bodies is a feature described in both humans [30] and atopic dogs [31]. Since lamellar bodies contain lipids and antimicrobial peptides, defects in secretion are associated not only with deficiencies in lipids but also with decreased antimicrobial peptides typical of atopic dermatitis [32].

Lipid abnormalities in atopic patients have been extensively documented in both people and dogs. In dogs, initial studies described decreased content of epidermal ceramides in atopic dogs (even non-lesional skin) compared with normal skin [33]. This decreased content was proposed to explain the impaired skin barrier of atopic dogs and the increased TEWL [34]. More recent studies have also reported a decrease in free fatty acid levels and a decreased ratio of ceramide [NS] C44/C34 in canine atopic skin [35]. These changes in lipid composition are responsible for an altered lamellar packing with a shift from orthorhombic to hexagonal arrangement. These alterations in the organization of the lamellae are important because the hexagonal packing is less dense compared with the orthorhombic and is associated with increased permeability. Similar alterations are described in atopic people, where ceramide deficiencies are well documented [36,37] and abnormal organization of the lipid lamellae shifting toward hexagonal packing is reported [38].

In horses, there are several older studies that have measured skin surface lipids in normal animals [39,40], but there is no published study on epidermal lipids in both normal and allergic horses. Thus, we do not know if allergic horses have altered epidermal ceramides, as reported in other species.

Another important player in skin barrier function and proper keratinization is filaggrin [41]. Filaggrin bioproducts compose the natural moisturizing factors; thus, any deficiency leads to decreased hydration of the skin and impaired skin barrier function [42]. Inflammation itself can affect filaggrin synthesis; thus, some of these changes are not necessarily primary but compounded once allergic inflammation develops [43]. In people, loss-of-function mutations in the filaggrin gene are one of the most reported risk factors for the development of atopic dermatitis [44,45]. Despite this, it is important to note that the presence of a mutation is neither sufficient nor necessary for the development of disease [46]. In dogs, mixed results have been reported on filaggrin. Some authors have reported increased filaggrin gene expression in atopic skin [47], while others reported a decrease [48]. Filaggrin mutations do not appear to play a major role in canine disease based on our current knowledge and may be greatly bred and country-dependent [49]. Currently, there is no published study on equine filaggrin, either in normal or in atopic skin; thus, the role of filaggrin in equine disease is completely unknown.

So far, there is no published report on antimicrobial peptides or skin microbiomes in allergic horses either. The microbiome of horses with pastern dermatitis has shown a decrease in bacterial alpha diversity in affected pasterns and a dysbiosis in favor of Staphylococcus [50]. The effect was correlated to the severity of clinical disease and prior antibacterial treatment [51]. Dysbiosis and predominance of Staphylococcus are reported in both allergic dogs [52,53] and people [54,55] as flares develop. Preliminary studies on intestinal [56] and upper and lower respiratory tract microbiota [57] in horses with asthma show differences from normal horses, but it is not known if this is a primary feature of allergic disease in horses or not.

Much of the research on transcriptomic changes in the skin of allergic horses has focused on insect bite hypersensitivity [58]. A study showed that lesional allergic skin has substantial transcriptomic differences when compared with healthy skin, namely downregulation of genes important to skin barriers like tight junctions, keratins, and upregulation of serine proteases, IL-13, and IL-5 receptor gene expression. These changes may be secondary to inflammation and not necessarily a primary feature of the disease. Of all TH2 cytokines, IL-13 gene expression was the main one found to be different. Of interest, genes important in terminal keratinocyte differentiation like filaggrin, involucrin, and loricrin showed high expression in lesional allergic skin and were comparable to normal skin. The authors concluded that in lesional allergic skin, there are alterations of adherens junctions but not a deficiency in terminal differentiation of keratinocytes, as reported in people. Non-lesional allergic skin had some changes but overall was quite similar to normal skin. When lesional allergic skin was compared with non-lesional allergic skin, downregulation of tight junction protein genes and upregulation of proteases and S100 protein genes were found. These changes are most likely due to inflammation rather than being primary differences in allergic skin. When the epidermis was specifically analyzed, important differences in gene expression were found between normal and non-lesional epidermis from horses diagnosed with insect bite hypersensitivity. Genes involved included genes involved in lipid metabolism (e.g., downregulation of enzymes involved in sphingolipid synthesis and downregulation of SCEL, the gene encoding for sciellin, a protein important for the assembly of cornified envelope). Upregulation of gene expression of the IL-31 receptor subunit was also found. These changes may be a “signature of disease” or due to “subclinical inflammation”.

**Figure 4:**
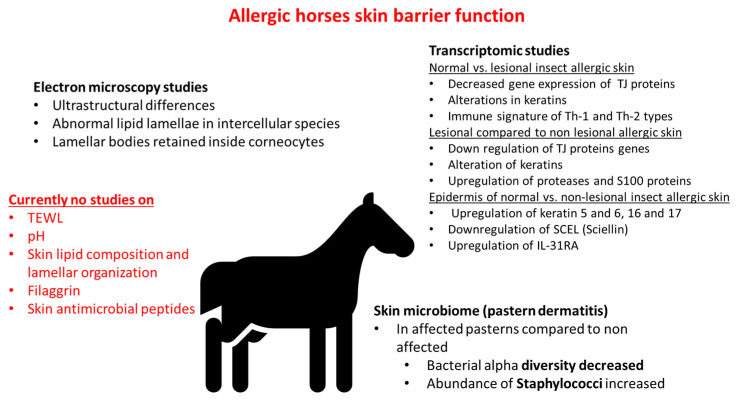
Summary of the current level of information on skin barrier parameters, microbiome, and transcriptomic changes in the skin of allergic horses. TJ: tight junctions; IL31-RA: IL-31 receptor alpha subunit.

In another study, the same research group reported on transcriptome differences between primary keratinocyte cultures of insect-allergic horses compared with healthy controls [59]. When keratinocytes were unstimulated, the only difference was in gene CTSL which encodes for cathepsin, which is a protease involved in cell migration. Transcriptomic differences between allergic and normal keratinocytes were also not very different after Culicoides stimulation. The most relevant differences between the two groups of keratinocytes were seen when keratinocytes were stimulated with cytokines like IL-4 and TNF α. The cytokine stimulation led to the downregulation of genes involved in the epithelial barrier and upregulation of immune response, indicating that the changes were a consequence of inflammation. Upregulation of genes encoding for chemokines was also noted. Importantly, keratinocytes had increased gene expression of IL-31 when stimulated with allergic cytokines. The response of normal and allergic keratinocytes was largely similar upon stimulation, with the exception being CXCL10 and CXCL11 upregulation in allergic but not in normal keratinocytes. This is the first report of equine keratinocytes being able to produce IL-31, although the study only reported on gene expression and not on protein synthesis. The study confirms that keratinocytes are capable of responding as amplifiers of the immune reaction when stimulated with cytokines, thus contributing to the recruitment of inflammatory cells and skin damage. One limitation of the study was that allergic keratinocytes were basal keratinocytes and that they were not harvested from lesional skin biopsies. These factors may explain the lack of changes in filaggrin gene expression. Moving forward, it will be beneficial to investigate changes in differentiated keratinocytes and study keratinocytes harvested from lesional allergic skin. The relevance of IL-31 in equine allergies and pruritus is being confirmed in various studies [60,61]. The current knowledge on skin barrier function in allergic horses is summarized in Figure 4. 

## 4. Conclusions

In summary, a few studies on skin ultrastructure in allergic horses seem to confirm similar changes characteristic of atopic dermatitis in other species (e.g., disorganized lipid lamellae, retained lamellar bodies in corneocytes). There is no information on whether these structural changes result in functional impairment (e.g., increased TEWL, increased pH) or whether the epicutaneous route of allergen exposure is as important for sensitization and elicitation of clinical signs in horses as it is in people and dogs. Transcriptomic studies of the epidermis of horses with insect bite hypersensitivity showed differences in gene expression between normal and non-lesional epidermis, such as downregulation of genes important for lipid metabolism and upregulation of gene expression of IL-31 receptor subunit. Equine keratinocytes respond to cytokine with stimulation by increasing gene expression of IL-31, highlighting the importance of this cytokine in equine allergic skin disease. Currently, it is unknown whether filaggrin is important in equine allergies, whether any difference in expression exists between normal and allergic horses, or whether changes are secondary to inflammation or a signature of disease. Clearly, more research is needed in horses on skin barrier function parameters. It could be of particular importance to study skin pH in allergic horses, since in other species, this parameter is one of the most reliable measurements of skin barrier function (less variability than TEWL measurements) and a critical factor for the maintenance of healthy homeostasis of the skin. An increase in pH is associated with skin infections like superficial pyoderma; thus, investigations of how this parameter changes in the course of allergy flares can prove to be very useful. Antibiotic resistance is a growing concern worldwide, and it is also affecting horses. Allergic horses are prone to recurrent bouts of Staphylococcal pyoderma; thus, studies on skin microbiome in the pathogenesis of equine skin allergies are very important. It is becoming clear that the way of the future is to restore biodiversity in the skin rather than rely on antibiotic strategies; thus, a thorough understanding of skin microbiome and bacterial diversity in equine allergic disease can inform future therapeutic options that could be more sustainable and less conducive to antibiotic resistance.

## Data Availability

No new data were created or analyzed in this study.

## References

[1] Jensen J.M., Proksch E. (2009). The skin’s barrier. G. Ital. Dermatol. Venereol..

[2] Rajkumar J., Chandan N., Lio P., Shi V. (2023). The Skin Barrier and Moisturization: Function, Disruption, and Mechanisms of Repair. Ski. Pharmacol. Physiol..

[3] Lee H.-J., Kim M. (2022). Skin Barrier Function and the Microbiome. Int. J. Mol. Sci..

[4] Harris-Tryon T.A., Grice E.A. (2022). Microbiota and maintenance of skin barrier function. Science.

[5] Verdier-Sévrain S., Bonté F. (2007). Skin hydration: A review on its molecular mechanisms. J. Cosmet. Dermatol..

[6] Proksch E. (2018). pH in nature, humans and skin. J. Dermatol..

[7] Schmuth M., Eckmann S., Moosbrugger-Martinz V., Ortner-Tobider D., Blunder S., Trafoier T., Gruber R., Elias P.M. (2024). Skin Barrier in Atopic Dermatitis. J. Investig. Dermatol..

[8] Santoro D., Saridomichelakis M., Eisenschenk M., Tamamoto-Mochizuki C., Hensel P., Pucheu-Haston C., International Committee on Allergic Diseases of Animals (ICADA) (2024). Update on the skin barrier, cutaneous microbiome and host defense peptides in canine atopic dermatitis. Vet. Dermatol..

[9] Yang G., Seok J.K., Kang H.C., Cho Y.-Y., Lee H.S., Lee J.Y. (2020). Skin Barrier Abnormalities and Immune Dysfunction in Atopic Dermatitis. Int. J. Mol. Sci..

[10] Sherenian M.G., Kothari A., Biagini J.M., Kroner J.W., Baatyrbek Kyzy A., Johannson E., Atluri G., He H., Martin L.J., Khurana Hershey G.K. (2021). Sensitization to peanut, egg or pets is associated with skin barrier dysfunction in children with atopic dermatitis. Clin. Exp. Allergy.

[11] Marsella R., Olivry T., Carlotti D., International Task Force on Canine Atopic Dermatitis (2011). Current evidence of skin barrier dysfunction in human and canine atopic dermatitis. Vet. Dermatol..

[12] Olivry T., Paps J.S., Amalric N. (2022). Transient and reversible reduction of stratum corneum filaggrin degradation products after allergen challenge in experimentally mite-sensitised atopic dogs. Vet. Dermatol..

[13] Beck L.A., Leung D.Y. (2000). Allergen sensitization through the skin induces systemic allergic responses. J. Allergy Clin. Immunol..

[14] Danby S.G., Cork M.J. (2018). pH in Atopic Dermatitis. Curr. Probl. Dermatol..

[15] Marsella R. (2024). Investigation into the Effects of Allergen Exposure and Topical Vinegar and Water Spray on Skin Barrier Parameters in Atopic Dogs. Vet. Sci..

[16] Peer R.P., Burli A., Maibach H.I. (2022). Unbearable transepidermal water loss (TEWL) experimental variability: Why?. Arch. Dermatol. Res..

[17] Green M., Feschuk A.M., Kashetsky N., Maibach H.I. (2022). “Normal” TEWL-how can it be defined? A systematic review. Exp. Dermatol..

[18] Alexander H., Brown S., Danby S., Flohr C. (2018). Research Techniques Made Simple: Transepidermal Water Loss Measurement as a Research Tool. J. Investig. Dermatol..

[19] Ali S.M., Yosipovitch G. (2013). Skin pH: From basic science to basic skin care. Acta Derm. Venereol..

[20] Cobiella D., Archer L., Bohannon M., Santoro D. (2019). Pilot study using five methods to evaluate skin barrier function in healthy dogs and in dogs with atopic dermatitis. Vet. Dermatol..

[21] Discepolo D., Gaare E., Handlos G., Perry E.B. (2024). Fluctuations in equine cutaneous pH and transepidermal water loss with time of day and ambient conditions. J. Equine Vet. Sci..

[22] Szczepanik M.P., Wilkołek P.M., Pluta M., Adamek Ł.R., Pomorski Z.J. (2012). The examination of biophysical parameters of skin (tran-sepidermal water loss, skin hydration and pH value) in different body regions of ponies. Pol. J. Vet. Sci..

[23] Szczepanik M., Wilkołek P., Pluta M., Adamek L.R., Gołyński M., Pomorski Z., Sitkowski W. (2013). The examination of biophysical skin parameters (transepidermal water loss, skin hydration and pH value) in different body regions in Polish ponies. Pol. J. Vet. Sci..

[24] Szczepanik M., Wilkołek P., Adamek Ł.R., Pluta M., Gołyński M., Sitkowski W., Kalisz G., Taszkun I., Pomorski Z.J. (2016). Influence of horse breed on transepidermal water loss. Pol. J. Vet. Sci..

[25] Cekiera A., Popiel J., Siemieniuch M., Jaworski Z., Slowikowska M., Siwinska N., Zak A., Niedzwiedz A. (2021). The examination of biophysical parameters of the skin in Polish Konik horses. PLoS ONE.

[26] Gołyński M., Szczepanik M., Wilkołek P., Adamek Ł.R., Gołyński M., Sitkowski W., Taszkun I. (2018). Influence of hair clipping on transepidermal water loss values in horses: A pilot study. Pol. J. Vet. Sci..

[27] O’shaughnessy-Hunter L.C., Yu A., Rousseau J.D., Foster R.A., Weese J.S. (2021). Longitudinal study of the cutaneous microbiota of healthy horses. Vet. Dermatol..

[28] Strompfová V., Štempelová L. (2024). Composition and diversity of 16S rRNA based skin bacterial microbiome in healthy horses. Vet. Res. Commun..

[29] Marsella R., Johnson C., Ahrens K. (2014). First case report of ultrastructural cutaneous abnormalities in equine atopic dermatitis. Res. Vet. Sci..

[30] Elias P.M., Wakefield J.S. (2014). Mechanisms of abnormal lamellar body secretion and the dysfunctional skin barrier in patients with atopic dermatitis. J. Allergy Clin. Immunol..

[31] Marsella R., Samuelson D., Doerr K. (2010). Transmission electron microscopy studies in an experimental model of canine atopic dermatitis. Vet. Dermatol..

[32] Rieg S., Steffen H., Seeber S., Humeny A., Kalbacher H., Dietz K., Garbe C., Schittek B. (2005). Deficiency of dermcidin-derived antimicrobial peptides in sweat of patients with atopic dermatitis correlates with an impaired innate defense of human skin in vivo. J. Immunol..

[33] Reiter L.V., Torres S.M.F., Wertz P.W. (2009). Characterization and quantification of ceramides in the nonlesional skin of canine patients with atopic dermatitis compared with controls. Vet. Dermatol..

[34] Shimada K., Yoon J.S., Yoshihara T., Iwasaki T., Nishifuji K. (2009). Increased transepidermal water loss and decreased ceramide content in lesional and non-lesional skin of dogs with atopic dermatitis. Vet. Dermatol..

[35] Chermprapai S., Broere F., Gooris G., Schlotter Y.M., Rutten V.P., Bouwstra J.A. (2018). Altered lipid properties of the stratum corneum in Canine Atopic Dermatitis. Biochim. Biophys. Acta Biomembr..

[36] Uchida Y., Park K. (2021). Ceramides in Skin Health and Disease: An Update. Am. J. Clin. Dermatol..

[37] van Smeden J., Bouwstra J.A. (2016). Stratum Corneum Lipids: Their Role for the Skin Barrier Function in Healthy Subjects and Atopic Dermatitis Patients. Curr. Probl. Dermatol..

[38] Pilgram G.S., Vissers D.C., van der Meulen H., Koerten H.K., Pavel S., Lavrijsen S.P., Bouwstra J.A. (2001). Aberrant lipid organization in stratum corneum of patients with atopic dermatitis and lamellar ichthyosis. J. Investig. Dermatol..

[39] Downing D.T., Vi S.W.C. (1980). Skin surface lipids of the horse. Lipids.

[40] Nicolaides N., Fu H.C., Rice G.R. (1968). The skin surface lipids of man compared with those of eighteen species of animals. J. Investig. Dermatol..

[41] Hoober J.K., Eggink L.L. (2022). The Discovery and Function of Filaggrin. Int. J. Mol. Sci..

[42] Kim Y., Lim K.-M. (2021). Skin barrier dysfunction and filaggrin. Arch. Pharmacal Res..

[43] Furue M. (2020). Regulation of Filaggrin, Loricrin, and Involucrin by IL-4, IL-13, IL-17A, IL-22, AHR, and NRF2: Pathogenic Implications in Atopic Dermatitis. Int. J. Mol. Sci..

[44] Palmer C.N.A., Irvine A.D., Terron-Kwiatkowski A., Zhao Y., Liao H., Lee S.P., Goudie D.R., Sandilands A., Campbell L.E., Smith F.J.D. (2006). Common loss-of-function variants of the epidermal barrier protein filaggrin are a major predisposing factor for atopic dermatitis. Nat. Genet..

[45] Kawasaki H., Kubo A., Sasaki T., Amagai M. (2011). Loss-of-function mutations within the filaggrin gene and atopic dermatitis. Curr. Probl. Dermatol..

[46] Moosbrugger-Martinz V., Leprince C., Méchin M.-C., Simon M., Blunder S., Gruber R., Dubrac S. (2022). Revisiting the Roles of Filaggrin in Atopic Dermatitis. Int. J. Mol. Sci..

[47] Theerawatanasirikul S., Sailasuta A., Thanawongnuwech R., Suriyaphol G. (2012). Alterations of keratins, involucrin and filaggrin gene expression in canine atopic dermatitis. Res. Vet. Sci..

[48] Roque J.B., O’Leary C.A., Kyaw-Tanner M., Duffy D.L., Shipstone M. (2011). Real-time PCR quantification of the canine filaggrin orthologue in the skin of atopic and non-atopic dogs: A pilot study. BMC Res. Notes.

[49] Wood S.H., Ollier W.E., Nuttall T., McEwan N.A., Carter S.D. (2010). Despite identifying some shared gene associations with human atopic dermatitis the use of multiple dog breeds from various locations limits detection of gene associations in canine atopic dermatitis. Vet. Immunol. Immunopathol..

[50] Kaiser-Thom S., Hilty M., Axiak S., Gerber V. (2021). The skin microbiota in equine pastern dermatitis: A case-control study of horses in Switzerland. Vet. Dermatol..

[51] Sangiorgio D.B., Hilty M., Kaiser-Thom S., Epper P.G., Ramseyer A.A., Overesch G., Gerber V.M. (2021). The influence of clinical severity and topical antimicrobial treatment on bacteriological culture and the microbiota of equine pastern dermatitis. Vet. Dermatol..

[52] Pierezan F., Olivry T., Paps J.S., Lawhon S.D., Wu J., Steiner J.M., Suchodolski J.S., Hoffmann A.R. (2016). The skin microbiome in allergen-induced canine atopic dermatitis. Vet. Dermatol..

[53] Bradley C.W., Morris D.O., Rankin S.C., Cain C.L., Misic A.M., Houser T., Mauldin E.A., Grice E.A. (2016). Longitudinal Evaluation of the Skin Microbiome and Association with Microenvironment and Treatment in Canine Atopic Dermatitis. J. Investig. Dermatol..

[54] Demessant-Flavigny A., Connétable S., Kerob D., Moreau M., Aguilar L., Wollenberg A. (2023). Skin microbiome dysbiosis and the role of *Staphylococcus aureus* in atopic dermatitis in adults and children: A narrative review. J. Eur. Acad. Dermatol. Venereol..

[55] Hwang J.B., Jaros J., Shi V.Y. (2020). *Staphylococcus aureus* in Atopic Dermatitis: Past, Present, and Future. Dermatitis.

[56] Leclere M., Costa M.C. (2020). Fecal microbiota in horses with asthma. J. Vet. Intern. Med..

[57] Bond S.L., Timsit E., Workentine M., Alexander T., Léguillette R. (2017). Upper and lower respiratory tract microbiota in horses: Bacterial communities associated with health and mild asthma (inflammatory airway disease) and effects of dexamethasone. BMC Microbiol..

[58] Cvitas I., Oberhänsli S., Leeb T., Dettwiler M., Müller E., Bruggman R., Marti E.I. (2020). Investigating the epithelial barrier and immune signatures in the pathogenesis of equine insect bite hypersensitivity. PLoS ONE.

[59] Cvitas I., Oberhaensli S., Leeb T., Marti E. (2022). Equine keratinocytes in the pathogenesis of insect bite hypersensitivity: Just another brick in the wall?. PLoS ONE.

[60] Craig N.M., Munguia N.S., Trujillo A.D., Chan A.M., Wilkes R., Dorr M., Marsella R. (2024). Interleukin 31 mediates pruritus in horses. Am. J. Vet. Res..

[61] Olomski F., Fettelschoss V., Jonsdottir S., Birkmann K., Thoms F., Marti E., Bachmann M.F., Kündig T.M., Fettelschoss-Gabriel A. (2020). Interleukin 31 in insect bite hypersensitivity—Alleviating clinical symptoms by active vaccination against itch. Allergy.

